# Life satisfaction of older adults and its influencing factors: an exploratory study of community-dwelling older adults in Xiamen, China

**DOI:** 10.3389/fpubh.2026.1699050

**Published:** 2026-01-30

**Authors:** Zhenping Zhang, Laifu Xiao, Yanlong Lin, Tianqi Sun

**Affiliations:** 1 International School, Huaqiao University, Quanzhou, China; 2 College of Education and People’s Livelihood, Xiamen City University, Xiamen, China; 3 Division of Aging Affairs, Xiamen Municipal Civil Affairs Bureau, Xiamen, China; 4 School of Civil Engineering, Huaqiao University, Xiamen, China

**Keywords:** aging population, economic security, older adults, health, life satisfaction, social support, Xiamen

## Abstract

**Introduction:**

The global population is aging rapidly, with China witnessing a significant growth in its older adult population. Understanding the life satisfaction of community-dwelling older adults and its determinants is crucial for informing age-friendly policies in rapidly aging urban areas.

**Methods:**

A cross-sectional survey was conducted among 2,225 community-dwelling adults aged 60 years and above in Xiamen, China, from March to May 2025. The survey collected data on demographics, economic status, health, social support, and environmental conditions. The primary outcome variable, “overall life satisfaction,” was measured using a single 5-point Likert scale (1 = very satisfied to 5 = very dissatisfied), with a “high level of life satisfaction” defined as a score of 1–2. A multinomial generalized linear model was used to identify influencing factors, and collinearity diagnostics (variance inflation factor, VIF) were performed to ensure model reliability.

**Results:**

A total of 91.5% of the participants reported a high level of life satisfaction. Key factors significantly influencing life satisfaction included self-rated health, daily physical and mental state, community-based social support, and economic factors (i.e., healthcare expenditure and financial support from children).

**Discussion:**

This study provides preliminary empirical evidence for formulating age-friendly policies in Xiamen and similar urban areas. Enhancing healthcare accessibility, improving community-based older adults support, and strengthening financial support systems may effectively improve life satisfaction among community-dwelling older adults. Future research should adopt a longitudinal design and include a broader range of older adult populations (e.g., institutionalized older adults) to deepen the understanding of how to best support the well-being of older adults in China.

## 1 Introduction

In recent decades, the global population has been experiencing a significant demographic shift toward aging. The number of people aged 60 and older worldwide is projected to increase from 1.1 billion in 2023 to 1.4 billion by 2030 (1). This trend is particularly evident and rapid in developing countries and regions. China, as the world’s second most populous country with the largest aging population, is no exception to this trend. The aging process in China has been accelerating, with the proportion of the older adults population (aged 60 and above) increasing steadily. By the end of 2024, the population aged 60 and over in China had reached 310 million, accounting for 22.0% of the national population, while those aged 65 and over numbered 220 million, or 15.6% (2). This demographic change has far-reaching implications for various aspects of society, including healthcare, social welfare, and economic development (3, 4, 29).

Life satisfaction is a crucial indicator of the well-being of the older adults (5–7). It reflects their overall subjective evaluation of their quality of life, which is influenced by multiple factors such as physical health, social relationships, economic status, and environmental conditions (7–10, 27, 28). High life satisfaction among the older adults is associated with better mental and physical health outcomes. For example, studies have shown that older adults individuals with higher life satisfaction are more likely to have lower levels of depression and anxiety (11), and they may also have a stronger immune system and better self-reported health (12). To systematically explore these influencing factors and their underlying mechanisms, we explicitly link each of the four guiding theoretical frameworks to the study’s analytical variables: Socio-economic Status Theory connects to healthcare expenditure, financial support from children, and per capita living space—proxies for material resource access, a core determinant of older adults life satisfaction per the theory; Social Support Theory guides the inclusion of emotional support from children, timely community support, and proactive community care, capturing the informal and formal support emphasized by the framework; Activity Theory informs variables like travel frequency and medicine forgetfulness (a proxy for health management engagement), reflecting meaningful activity’s role in buffering age-related well-being decline; and the Socio-ecological Model underpins the integration of individual variables (self-rated health, chronic diseases) and environmental variables (living arrangement, community services), reflecting the theory’s focus on individual-context interplay.

Xiamen, a coastal city in southeast China, serves as an interesting case for the study of older adults life satisfaction. As a developed city, Xiamen has witnessed a continuous growth in its older adults population in recent years. As of the end of 2024, there were 643,000 residents aged 60 and above in Xiamen, representing 12.02% of the city’s permanent population, of whom 474,000 were registered permanent residents, accounting for 15.3% of the city’s registered population (13). It has distinct urban – rural characteristics (14). In urban areas, there are modern high-rise buildings, well-developed public transportation systems, and a wide range of cultural and recreational facilities. However, rural areas in Xiamen also coexist with unique local traditions, closer-knit community relationships, and relatively lower-cost living environments.

National-level evidence on older adults’ life satisfaction in China has overwhelmingly relied on the China Health and Retirement Longitudinal Study (CHARLS), the China Family Panel Studies (CFPS), the CGSS (Chinese General Social Survey) and the Survey on the Living Conditions of China’s Urban and Rural Older Persons. These studies consistently show that self-rated health, pension adequacy, and inter-generational support are significant predictors of subjective well-being (4, 15). However, their macro-level focus obscures regional heterogeneity produced by diverging demographic structures, welfare regimes, and service landscapes (16). City-specific investigations are still scarce and geographically skewed toward mega-cities such as Beijing, Shanghai, and Guangzhou. Strikingly, no peer-reviewed English article has examined life satisfaction among older adults in Xiamen—an economically vibrant, rapidly aging coastal city with a distinct Minnan culture. The single extant study used a small sample (*n* = 197) and the WHOQOL-BREF, but did not isolate life satisfaction or test policy-relevant variables (14). Consequently, there is a clear need for an exploratory study that identifies context-specific determinants of life satisfaction among Xiamen’s older adults residents.

Compared to the proportion of community-dwelling older adults, the rate of older adults individuals living in nursing homes in Xiamen is inherently low, typically between 1 and 1.5%. According to statistics from Xiamen Municipal Bureau of Civil Affairs, as of August 2025, 6,554 older adults residents live in nursing homes, accounting for 1.29% of the city’s 507,000 registered older adults population. The central objective of this study is to investigate the current level of life satisfaction among community-dwelling adults aged 60 and above in Xiamen and to identify the key factors that shape their subjective well-being. Specifically, the research seeks to answer two interrelated questions. First, what is the overall level of life satisfaction reported by Xiamen’s community-dwelling older residents? Second, which demographic, socio-economic, health-related, social-relational, and environmental variables most strongly affect variations in life satisfaction within this rapidly aging coastal city?

The remainder of the paper is organized as follows. Section 2 reviews the theoretical and empirical literature on determinants of life satisfaction in later life. Section 3 describes the research design and data collection procedures. Section 4 presents descriptive statistics and multivariate findings. Section 5 discusses the implications of the results, research limitations, and avenues for future research. Section 6 summarizes key findings and conclusions.

## 2 Literature review

### 2.1 Life satisfaction and influencing factors

Life satisfaction is broadly defined as a global, cognitive judgment of one’s overall quality of life (6, 7, 17). Unlike momentary affect, this appraisal reflects a long-term, integrative evaluation across life domains such as health, family, income, and neighborhood conditions (5, 18). Among older adults, four complementary theoretical frameworks dominate empirical research.

First, Socio-economic Status Theory links income, education, and occupational prestige to differential access to material resources and health services, thereby shaping life satisfaction (11). A large-scale study of Shanghai elders demonstrated that perceived relative income was the strongest predictor of life satisfaction, whereas absolute income mattered only when basic needs were unmet (16).

Second, Social Support Theory emphasizes the protective role of both structural (e.g., frequency of contact with children) and functional (e.g., emotional and financial assistance) support in later life (19–21, 31, 32, 34). Using 2020 China Family Panel Study (CFPS) data, Shen et al. (15) found that informal support—especially frequent communication with children—explained more variance in life satisfaction than formal pension coverage, underscoring the primacy of family ties in collectivist contexts.

Third, Activity Theory posits that continued engagement in meaningful activities—whether productive, social, or leisure—buffers age-related losses and sustains psychological well-being (22, 23, 30, 33). Recent Chinese evidence shows that older adults who participate in community sports clubs or volunteer groups report significantly higher life satisfaction than their less active peers (24). Another research also shows that friendly conversation has significant positive relationships with life satisfaction (5).

Finally, Socio-ecological models integrate physical and social neighborhood environments as important determinants. Zhang and Li’s (24) Nanjing study revealed that perceived neighbor support, availability of exercise facilities, and sidewalk quality indirectly enhanced life satisfaction by fostering social capital and physical activity. Similar pathways were confirmed in Shanghai, where cognitive social capital (trust and reciprocity) mediated the effect of community health-care accessibility on life satisfaction for older women, but not for older men (16).

Across frameworks, gender and rural–urban disparities are recurrent moderators. Female elders benefit more from emotional support and community trust, whereas male elders derive greater satisfaction from income security and instrumental support (15). Rural elders place higher value on medical insurance and family financial transfers, whereas urban elders prioritize recreational facilities and neighborhood safety (24). These findings underscore the need for context-specific measures when assessing life satisfaction in rapidly aging Chinese cities such as Xiamen.

### 2.2 Studies on older adults life satisfaction in China and Xiamen

Recent studies on older adults life satisfaction in China converge on three core determinants—health, economic security, and social embeddedness—while simultaneously revealing marked regional heterogeneity. Analyses of the 2011/2012 Chinese Longitudinal Healthy Longevity Survey (CLHLS) show that self-rated health and functional independence are the strongest predictors, followed by perceived relative economic status and access to social-security provisions (9). In urban Shanghai, perceived neighborhood safety and cognitive social capital—trust, reciprocity, and a sense of belonging—mediate the effect of community resources on life satisfaction, with gender moderating these pathways: older women benefit more from informal social cohesion, whereas older men derive greater satisfaction from formal civic participation (16). City-specific work in Nanjing further demonstrates that neighbor support and accessibility to daily-life facilities exert independent, positive effects on multiple domains of quality of life (24).

Rural–urban comparisons highlight contextual specificity: urban elders’ well-being is closely tied to accessible healthcare and transport, while rural elders’ satisfaction hinges on medical-insurance coverage rather than pension generosity (15). Turning to Xiamen, the evidence base remains thin. Sun et al. (14) surveyed 197 community-dwelling elders in 2017 but relied on descriptive statistics and basic multivariate tests.

## 3 Research methodology

A quantitative, cross-sectional survey design was adopted to capture the life satisfaction of community-dwelling older residents in Xiamen from March to May 2025. The study is exploratory in nature; its primary aim is to identify and quantify the associations between life satisfaction and a set of theoretically derived predictors drawn from Socio-economic Status Theory, Social Support Theory, Activity Theory and Socio-ecological models. The questionnaire was adapted from the Survey on the Living Conditions of China’s Urban and Rural Older Persons (developed by the National Working Committee on Aging of China)—a widely used instrument. We made minor adjustments to fit Xiamen’s local context (e.g., adding community service-related items) while preserving the original scale’s core structure.

As Table 1 shows, the questionnaire was structured around seven substantive domains to capture the full spectrum of determinants of life satisfaction among Xiamen’s older adults. Demographics (Items 1–4) capture core socio-structural backgrounds: gender (Item 1), five-year age bands (Item 2, for functional aging gradients), marital status (Item 3, as a proxy for household stability/emotional intimacy), and educational attainment (Item 4, indicating cognitive resources and socioeconomic trajectories). Economic Status (Items 5–9) maps income sources (Items 5–6: pensions, children’s transfers, etc., distinguishing absolute income from financial security) and key expenditures (Items 7–9: daily living, health care, leisure, to test satisfaction drivers like income sufficiency or expenditure burden).

**Table 1:** Summary of data collection instrument dimensions.

Module	No. of items	Key content
Demographics	4	Gender, age band, marital status, education level
Economic status	5	Main income sources (pension, children, savings, etc.), stable monthly income, monthly expenditures on daily living, health care, and leisure
Housing and environment	3	Living arrangement, per-capita floor area, overall satisfaction with community environment (sanitation, safety, amenities)
Health	6	Self-rated health, ADL dependency, chronic disease counts, frequency of preventive check-ups, medication adherence, daily physical and mental state
Social and activity participation	6	Smartphone/ICT ownership, short-video usage, frequency of physical exercise, participation in community-organized vs. peer-initiated activities, out-of-town travel
Social support	4	Contact frequency with children, adequacy of children’s financial support, adequacy of children’s emotional support, community assistance and proactive care
Life satisfaction	5	Overall life satisfaction, satisfaction with family/children support, transport convenience, older adults-care services, medical services

Housing and Environment (Items 10–12) includes living arrangements (Item 10, reflecting social ecology), per-capita floor area (Item 11, for material comfort/crowding), and a 5-point neighborhood satisfaction scale (Item 12, synthesizing cleanliness, safety, etc.). Health (Items 13–18) builds a multidimensional profile: self-rated health (Item 13), Activities of Daily Living (Item 14), chronic disease count (Item 15), health-care utilization (Items 16–17: check-ups, medication adherence), and physical/mental state appraisal (Item 18). The dependent variable Life Satisfaction (Items 30–34) uses five 5-point single-item scales: overall satisfaction (Item 30) and domain-specific satisfaction (family support, transport, community care, medical services; Items 31–34), enabling both overall scoring and targeted policy intervention insights.

Data collection was embedded within Xiamen’s established community-home care infrastructure. In accordance with the municipal policy “Notice on Further Improving Community-Based Home Care Services” (25), the city had procured professional care services and created frontline “elder-care assistants”—with a total of 1,050 such assistants evenly distributed across all urban communities and rural villages by administrative division, at a ratio of one assistant per 400 older residents. To ensure sampling rigor, we randomly selected 500 assistants from this full pool, covering diverse administrative regions (Siming, Huli, Jimei, Haicang, Tong’an, and Xiang’an). These selected assistants, who already providing daily outreach, health management and recreational services, were mobilized and trained to act as field interviewers. Each was instructed to collect data from 4 to 5 older adults, following the actual proportion of community-dwelling older adults in their respective areas to avoid exclusion bias. Leveraging their existing rapport with older adults, assistants conducted face-to-face interviews between March and May 2025, with the random selection of assistants and controlled per-person data quota ensuring geographic and socio-economic representativeness. The study was formally approved by the civil department of Xiamen. For participants with limited literacy, verbal consent (witnessed by community staff or family members) was obtained and documented; all participants were informed of their right to withdraw at any time without penalty. After data cleaning, 2,225 valid questionnaires were retained.

## 4 Results

### 4.1 Sample characteristics

A total of 2,225 community-dwelling older in Xiamen completed the survey. As shown in Table 2, the sample is predominantly female (61.57%), with more than half (55.06%) aged below 70 years. Nearly one-fifth are widowed (20.13%), and two-thirds have completed junior high school or less (66.56%). These figures closely mirror the city-wide demographic profile of older residents. It is noted that the total of the percentages may not add up to 100% due to rounding.

**Table 2:** Basic characteristics of the sample (*N* = 2,225).

Item		Frequency	Percentage (%)
Gender	Male	855	38.43
	Female	1,370	61.57
Age	60–64 years	659	29.62
	65–69 years	566	25.44
	70–74 years	430	19.33
	75–79 years	241	10.83
	80–84 years	193	8.67
	85 + years	136	6.11
Marital status	Married	1,702	76.49
	Widowed	448	20.13
	Divorced	50	2.25
	Never married	25	1.12
Education	No formal schooling	374	16.81
	Primary school	654	29.39
	Junior high school	453	20.36
	Senior high/technical school	425	19.10
	College	202	9.08
	Bachelor’s degree	100	4.49
	Postgraduate	17	0.76

### 4.2 Descriptive statistics of life satisfaction and influencing factors

The overall life satisfaction among the older population in Xiamen is relatively high, with 91.5% of the older adults individuals reporting being either very satisfied or fairly satisfied. However, 7.60% feel neutral about their living conditions, while only a small percentages report dissatisfaction, with 0.89% indicating they are not too satisfied or very dissatisfied. Satisfaction with family harmony and children’s support is notably high, with 91.24% expressing satisfaction. Transportation convenience also enjoys high satisfaction rates, with 88.86% of the older adults population being content. However, there is room for improvement in older adults care services and medical services, where 75.51 and 79.33% respectively, are satisfied, with 13.17% of the older adults not being aware of services like meal assistance, bathing help, and escort services, and 2.79% finding transportation inconvenient or very inconvenient (Table 3).

**Table 3:** Life satisfaction of the older adults in Xiamen (*N* = 2,225).

Item	Satisfaction Level	Frequency	Percentage (%)
Overall life satisfaction	Very satisfied	1,180	53.03
	Relatively satisfied	856	38.47
	Average	169	7.60
	Relatively dissatisfied	15	0.67
	Very dissatisfied	5	0.22
Satisfaction with family harmony and children’s support	Very satisfied	1,298	58.34
	Relatively satisfied	732	32.9
	Average	172	7.73
	Relatively dissatisfied	18	0.81
	Very dissatisfied	5	0.22
Satisfaction with the convenience of daily transportation	Very convenient	1,203	54.07
	Relatively convenient	774	34.79
	Average	186	8.36
	Relatively inconvenient	46	2.07
	Very inconvenient	16	0.72
Overall satisfaction with aged care services	Very satisfied	1,019	45.8
	Relatively satisfied	661	29.71
	Average	230	10.34
	Relatively dissatisfied	19	0.85
	Very dissatisfied	3	0.13
	Unaware	293	13.17
Overall satisfaction with medical services	Very satisfied	878	39.46
	Relatively satisfied	887	39.87
	Average	394	17.71
	Relatively dissatisfied	58	2.61
	Very dissatisfied	8	0.36

“Unaware” denotes no knowledge of the service, which is differentiated from the “Used” category to avoid misclassification bias in regression models.

In terms of economic income, pensions and financial support from children are the most prominent sources. Pensions account for 90.20% and financial support from children makes up 23.33%. Followed by labor income, savings deposits with interest earnings, and government subsidies, which are relatively comparable, accounting for 7.82, 6.07, and 4.58%, respectively. For the monthly stable economic income, the majority, 64.45% of the older adults, have an income ranging from 1,000 to 5,000 yuan. Additionally, 21.17% of the older adults have a monthly stable income below 1,000 yuan. Regarding expenditures, the distribution across different ranges for daily necessities (food, clothing, housing, transportation) is relatively even. Expenditures between 500 and 2,000 yuan account for 49.07%, while only 1.84% have expenditures exceeding 5,000 yuan. For medical and health expenditures, 56.90% of the older adults spend less than 500 yuan, and 95.15% have expenditures below 2,000 yuan, indicating that most older adults people have relatively good health and low medical expenses. Compared to daily and medical expenditures, spending on leisure and entertainment is relatively low. 67.64% of the older adults spend less than 500 yuan per month, and only 12.00% have monthly expenditures above 1,000 yuan (Table 4).

**Table 4:** Economic status of the older adults in Xiamen (*N* = 2,225).

Item		Frequency	Percentage (%)
Main sources of income (multiple answers allowed)	Pension	2007	90.20
	Support from children	519	23.33
	Labor income	174	7.82
	Savings and interest	135	6.07
	Government subsidy	102	4.58
	Investment/stocks	42	1.89
	Others	29	1.30
Stable monthly income	<CNY 500	241	10.83
	CNY 501–1,000	230	10.34
	CNY 1001–2000	426	19.15
	CNY 2001–3,000	411	18.47
	CNY 3001–5,000	597	26.83
	CNY 5001–8,000	212	9.53
	>CNY 8001	108	4.85
Monthly basic living expenses	<CNY 500	516	23.19
	CNY 501–1,000	535	24.04
	CNY 1001–2000	557	25.03
	CNY 2001–3,000	388	17.44
	CNY 3001–5,000	188	8.45
	CNY 5001–8,000	33	1.48
	>CNY 8001	8	0.36
Monthly medical and health expenses	<CNY 500	1,266	56.90
	CNY 501–1,000	619	27.82
	CNY 1001–2000	232	10.43
	CNY 2001–3,000	62	2.79
	CNY 3001–5,000	27	1.21
	CNY 5001–8,000	12	0.54
	>CNY 8001	7	0.31
Monthly leisure and recreational expenses	<CNY 500	1,505	67.64
	CNY 501–1,000	453	20.36
	CNY 1001–2000	167	7.51
	CNY 2001–3,000	65	2.92
	CNY 3001–5,000	22	0.99
	>CNY 5001	13	0.58

The vast majority (88.23%) of Xiamen community-dwelling older adults live with a spouse or children, while 9.71% live alone, 1.80% reside with other relatives/caregivers, and only 0.27% stay in community-embedded nursing institutions. Housing space is generous for most: 60.32% enjoy more than 30 m^2^ per capita, whereas 16.40% live in less than 20 m^2^ per person. Correspondingly, 85.17% express satisfaction with their living environment, 12.99% rate it as average, and 1.84% feel dissatisfied or very dissatisfied (Table 5).

**Table 5:** Housing and environment of the older adults in Xiamen (*N* = 2,225).

Item		Frequency	Percentage (%)
Living arrangement	Living alone	216	9.71
	Living with spouse	1,033	46.43
	Living with children	930	41.80
	Living with relatives/friends/caregivers	40	1.80
	Living in community-embedded nursing institution	6	0.27
Per-capita living space	>40 m^2^	819	36.81
	30–40 m^2^	523	23.51
	20–30 m^2^	518	23.28
	10–20 m^2^	328	14.74
	<10 m^2^	37	1.66
Satisfaction with overall living environment	Very satisfied	968	43.51
	Relatively satisfied	927	41.66
	Average	289	12.99
	Not very satisfied	32	1.44
	Very dissatisfied	9	0.40

Health status is the cornerstone of quality of life for older adults. Nearly two-thirds (66.87%) rate their health as “good” or “very good,” while 27.55% say it is “average” and 5.57% consider it “poor” or “very poor.” Functional independence is high: 84.72% are fully self-sufficient in daily activities, 12.04% need occasional help, and only 3.24% require frequent assistance or are completely dependent. Chronic-disease prevalence is moderate; cardiovascular, musculoskeletal and metabolic conditions dominate (35.19, 22.47 and 15.78% respectively). Remarkably, 36.40% report no chronic illness at all. Preventive-care habits are encouraging—66.34% undergo a physical check-up at least once a year—and medication adherence is acceptable, with 59.51% rarely or never forgetting doses. Overall, 75.10% describe their mental well-being as positive or cheerful, whereas 5.39% report persistent low mood or exhaustion (Table 6).

**Table 6:** Health status of the older adults in Xiamen (*N* = 2,225).

Item		Frequency	Percentage (%)
Self-rated health	Very good	648	29.12
	Good	840	37.75
	Average	613	27.55
	Poor	105	4.72
	Very poor	19	0.85
ADL independence	Fully self-sufficient	1885	84.72
	Needs occasional help	268	12.04
	Needs frequent help	52	2.34
	Completely dependent	20	0.90
Chronic diseases (multiple answers)	Cardiovascular disease	783	35.19
	Metabolic disease	351	15.78
	Musculoskeletal disease	500	22.47
	Neurological disease	31	1.39
	Respiratory disease	51	2.29
	Urological disease	89	4.00
	Ophthalmic disease	179	8.04
	Other chronic disease	231	10.38
	No chronic disease	810	36.40
Physical-exam frequency	At least once a year	1,476	66.34
	Every 1–2 years	456	20.49
	Every 3–5 years	146	6.56
	None in past 5 years	147	6.61
Medication adherence	Often forget	183	8.22
	Occasionally forget	718	32.27
	Rarely forget	511	22.97
	Never forget	813	36.54
Mental/emotional state	Energetic and cheerful	814	36.58
	Generally good, occasional fatigue	857	38.52
	Calm, sometimes bored	434	19.51
	Frequent fatigue/low mood	97	4.36
	Persistent exhaustion/depression	23	1.03

Smart-device usage is high among Xiamen seniors: 80.94% use a smartphone regularly and 43.24% watch short-form videos every day, yet 17.98% either lack smart devices or seldom use them. Physical activity is also common—34.70% exercise almost daily—while 27.91% rarely do. Participation in organized activities is fairly balanced: about one-third of respondents “often” or “occasionally” join events arranged by communities, friends, or senior centers, whereas roughly one-fifth “seldom” or “never” take part. Travel outside Xiamen is modest; only 39.64% leave the city once or more per year, 34.02% do so occasionally, and 26.34% almost never travel (Table 7).

**Table 7:** Social and activity participation of the older adults in Xiamen (*N* = 2,225).

Item		Frequency	Percentage %
Smart devices used regularly (multiple answers)	Smartphone	1801	80.94
	Tablet	159	7.15
	Desktop/laptop	140	6.29
	Smart watch/band	110	4.94
	Other wearables	22	0.99
	None/seldom use any	400	17.98
Frequency of watching short videos	Every day	962	43.24
	Often	437	19.64
	Occasionally	387	17.39
	Rarely	439	19.73
Frequency of physical exercise	Almost daily	772	34.70
	2–3 times/week	452	20.31
	Once a week	226	10.16
	1–2 times/month	154	6.92
	Rarely/never	621	27.91
Participation in neighborhood activities	Often	696	31.28
	Occasionally	697	31.33
	Rarely	455	20.45
	Never	377	16.94
Participation in friend−/institution-organized activities	Often	670	30.11
	Occasionally	698	31.37
	Rarely	387	17.39
	Never	470	21.12
Frequency of out-of-town travel	≥6 times/year	129	5.80
	5–6 times/year	88	3.96
	3–4 times/year	246	11.06
	1–2 times/year	419	18.83
	Occasionally	757	34.02
	Rarely/never	586	26.34

Family and community ties provide strong safety nets for Xiamen’s older adults. Nearly 7 in 10 elders (68.18%) either live with their children or speak with them daily, and another quarter (24.04%) connect once or twice a week. When financial needs arise, 73.3% report receiving frequent or occasional monetary support from their children, while only 7.91% seldom or never do. Emotional support is even more prevalent: 84.18% say they can “often” or “occasionally” count on children for companionship or comfort, and a mere 4.13% rarely or never receive such support. Beyond the family circle, the local neighborhood committee or village office is also responsive: 72.77% of elders can readily obtain help in times of trouble, and 76.45% are proactively contacted—by phone, WeChat, or home visits—at least a few times a year (Table 8).

**Table 8:** Social support of the older adults in Xiamen (*N* = 2,225).

Item		Frequency	Percentage %
Contact frequency with children	Live together/daily contact	1,517	68.18
	1–2 times per week	535	24.04
	1–2 times per month	91	4.09
	A few times per year or less	24	1.08
	Almost never	58	2.61
Financial support from children	Often/substantial support	1,185	53.26
	Occasional/moderate support	446	20.04
	Rare/limited support	98	4.40
	Almost none	78	3.51
	Never needed	418	18.79
Emotional support from children	Often/substantial support	1,468	65.98
	Occasional/moderate support	405	18.20
	Rare/limited support	63	2.83
	Almost none	29	1.30
	Never needed	260	11.69
Timely help from community/village	Often receive help	1,298	58.34
	Occasionally receive help	321	14.43
	Rarely receive help	48	2.16
	Almost never	59	2.65
	Never needed	499	22.43
Proactive care from community/village	Almost every month	1,220	54.83
	3–5 times per year	481	21.62
	1–2 times per year	298	13.39
	Rarely/never	122	5.48
	Prefer no contact	104	4.67

“Never needed” denotes awareness but no demand, “Prefer no contact” means personal preferences, both differentiated from the “frequency” category to avoid misclassification bias in regression models.

### 4.3 Bivariate associations and collinearity analysis

To examine the bivariate associations between each independent variable and life satisfaction, Kruskal-Wallis H tests were conducted. The results revealed that gender (Kruskal–Wallis *H* = 3.45, *p* = 0.06), age group (*H* = 5.09, *p* = 0.41), marital status (*H* = 6.45, *p* = 0.09), stable monthly income (*H* = 8.75, *p* = 0.19), monthly expenditure on leisure, etc. (*H* = 5.78, *p* = 0.33), living arrangement (*H* = 9.02, *p* = 0.06), and usage of smart devices (*H* = 0.83, *p* = 0.36) had no statistically significant associations with life satisfaction.

In contrast, education level (*H* = 45.13, *p* < 0.001), monthly expenditure on food, etc. (*H* = 59.36, *p* < 0.001), healthcare expenditure (*H* = 46.7, *p* < 0.001), per capita living space (*H* = 53.43, *p* < 0.001), neighborhood environment satisfaction (*H* = 883.78, *p* < 0.001), self-rated health (*H* = 480.08, *p* < 0.001), daily living ability (*H* = 57.11, *p* < 0.001), presence of chronic diseases (*H* = 42.59, *p* < 0.001), and regular physical check-ups (*H* = 48.82, *p* < 0.001), forgetfulness about medicine (*H* = 69.04, *p* < 0.001) were all significantly related to life satisfaction.

Additionally, variables related to lifestyle and social engagement, such as daily physical/mental state (*H* = 240.2, *p* < 0.001), frequency of watching short videos (*H* = 30.69, *p* < 0.001), participation in physical exercises (*H* = 27.02, *p* < 0.001), participation in community activities (*H* = 78.23, *p* < 0.001), social activities with friends (*H* = 72.73, *p* < 0.001), frequency of traveling outside Xiamen (*H* = 39.41, *p* < 0.001), frequency of contacting children (*H* = 45.21, *p* < 0.001), financial support from children (*H* = 248.31, *p* < 0.001), emotional support from children (*H* = 268.68, *p* < 0.001), timeliness of community support (*H* = 374.29, *p* < 0.001), proactivity of community care (*H* = 358.78, *p* < 0.001), transport convenience satisfaction (*H* = 968.42, *p* < 0.001), family harmony satisfaction (*H* = 824.98, *p* < 0.001), elder-care service satisfaction (*H* = 900.37, *p* < 0.001), and medical service satisfaction (*H* = 918.43, *p* < 0.001) also demonstrated significant relationships with life satisfaction. These findings indicate that a wide range of socio-demographic, economic, health, lifestyle, and environmental factors, collectively influence life satisfaction among the older adults in this study (Table 9).

**Table 9:** Bivariate associations between independent variables and life satisfaction.

Variable	Kruskal–Wallis *H*	Asymptotic significance
Gender	3.45	0.06
Age group	5.09	0.41
Marital status	6.45	0.09
Education level	45.13	0
Stable monthly income	8.75	0.19
Monthly expenditure on food, etc.	59.36	0
Healthcare expenditure	46.70	0
Monthly expenditure on leisure, etc.	5.78	0.33
Living arrangement	9.02	0.06
Per capita living space	53.43	0
Neighborhood environment satisfaction	883.78	0
Self-rated Health	480.08	0
Daily living ability	57.11	0
Presence of chronic diseases	42.59	0
Regular physical check-ups	48.82	0
Forgetfulness about medicine	69.04	0
Daily physical/mental state	240.20	0
None/seldom use any smart devices	0.83	0.36
Frequency of watching short videos	30.69	0
Participation in physical exercises	27.02	0
Participation in community activities	78.23	0
Social activities with friends	72.73	0
Frequency of traveling outside Xiamen	39.41	0
Frequency of contacting children	45.21	0
Financial support from children	248.31	0
Emotional support from children	268.68	0
Timeliness of community support	374.29	0
Proactivity of community care	358.78	0
Transport convenience satisfaction	968.42	0
Family harmony satisfaction	824.98	0
Elder-care service satisfaction	900.37	0
Medical service satisfaction	918.43	0

Prior to the regression analysis, collinearity diagnostics were conducted to evaluate potential multicollinearity among independent variables, with life satisfaction as the dependent variable. The variance inflation factor (VIF) was adopted as the core assessment indicator, and the detailed results are presented in Table 10. The VIF values of all independent variables ranged from 1.09 to 2.85, which are significantly below the critical threshold of 10 (a standard indicator for severe multicollinearity) and mostly below 5 (a threshold for moderate multicollinearity). Correspondingly, the tolerance values (reciprocal of VIF) for all variables were greater than 0.35 (calculated as 1/2.85), exceeding the critical value of 0.1 for severe multicollinearity. These results confirm that there is no severe multicollinearity among the independent variables in the regression model. The linear correlations between variables are weak, which ensures the stability and reliability of the subsequent regression coefficient estimates. Therefore, all included independent variables can be retained for the multiple linear regression analysis.

**Table 10:** Collinearity analysis.

Variable	VIF
Gender	1.09
Age group	1.52
Marital status	1.20
Education level	1.84
Stable monthly income	1.86
Monthly expenditure on food, etc.	1.90
Healthcare expenditure	1.52
Monthly expenditure on leisure, etc.	1.52
Living arrangement	1.21
Per capita living space	1.15
Neighborhood environment satisfaction	1.70
Self-rated Health	2.10
Daily living ability	1.53
Presence of chronic diseases	1.33
Regular physical check-ups	1.18
Forgetfulness about medicine	1.22
Daily physical/mental state	1.72
None/seldom use any smart devices	1.91
Frequency of watching short videos	1.85
Participation in physical exercises	1.70
Participation in community activities	2.73
Social activities with friends	2.85
Frequency of traveling outside Xiamen	1.61
Frequency of contacting children	1.41
Financial support from children	1.21
Emotional support from children	1.55
Timeliness of community support	1.54
Proactivity of community care	1.71
Transport convenience satisfaction	1.92
Family harmony satisfaction	1.68
Elder-care service satisfaction	2.40
Medical service satisfaction	2.34

### 4.4 Regression analysis

Because the outcome—life satisfaction is recorded on an ordered five-point scale, we initially specified an ordinal logistic regression. The test of parallel lines, however, decisively rejected the proportional-odds assumption (*p* < 0.001), implying that the effect of each predictor was not uniform across the satisfaction cut-points. To respect the ordinal nature of the outcome while accommodating these unequal odds, we adopted a generalized linear model (GLM). Specifically, we fitted a multinomial GLM with a multinomial distribution and a logit link function. This specification relaxes the proportional-odds constraint, permits category-specific effects for every predictor, and produces interpretable odds ratios that compare each level of satisfaction with the reference category.

Let Y ∈ {1,…,*k*} denote the ordered life-satisfaction levels (*k* = 5), and let x be the vector of predictors.

With category *k* as the reference, the model is


g_j(x)=log[P(Y=j∣x)/P(Y=k∣x)]=η_j(x),j=1,…,k–1

where

η_j(x) = β_{0j} + xᵀβ_j,

β_{0j} = intercept for category j,

β_j = coefficient vector for category j.

Using a multinomial generalized linear model, we examined how various factors shape the likelihood of older adults descending from “very satisfied” to “very dissatisfied” with life in Xiamen.

We first examined how satisfaction within five specific sub-dimensions affects overall life satisfaction (Model I). Due to the fact that the last option for older adults care service satisfaction (“Unaware”) does not represent a category of satisfaction but is more akin to a general evaluation, it has been modified to reflect a general sentiment, i.e., “Unaware” is modified to “average.” As Table 11 demonstrates, the model achieves a favorable fit: the chi-square value of 1985.92 (with 35 degrees of freedom) and a deviance/df ratio of 0.34 (well below 1) collectively indicate that the model explains the data effectively, with strong statistical significance.

**Table 11:** Model fit (*N* = 2,225).

Model	Chi-square	df	Deviance/df	AIC	BIC
Model I	1985.92***	35	0.34	2125.37	2347.96
Model II	1214.79***	100	0.34	3207.59	3801.17

*p < 0.05, **p < 0.01, and ***p < 0.001.

The regression analysis of Model I (*n* = 2,225) aligns with and extends insights from four theoretical frameworks, with “very dissatisfied” as the reference category for satisfaction-related variables. First, compared with Socio-economic Status Theory, which links resources like income and education to life satisfaction, we observed that while education level (Wald *χ*^2^ = 1.42, No significance) and age group (Wald *χ*^2^ = 2.82, No significance) did not exert significant effects, marital status (odds ratio range: 2.82–3.27, No significance) and gender [Exp(B) = 1.19, No significance] hinted at potential socio-economic nuances—though these were not statistically significant. Second, in line with Social Support Theory, which emphasizes the protective role of family and social ties, family harmony satisfaction emerged as a dominant predictor [Wald *χ*^2^ = 187.21, *p* < 0.001, Exp(B) = 0.02]. This indicates that individuals “very satisfied” with family harmony have only 0.02 times the odds of low life satisfaction relative to those “very dissatisfied,” underscoring the critical role of familial support. Third, while Activity Theory, which highlights the value of meaningful engagement, was not directly measured via activity participation in this model, proxies like transport convenience satisfaction [Wald *χ*^2^ = 56.65, *p* < 0.001, Exp(B) = 0.19] and medical service satisfaction [Wald *χ*^2^ = 57.22, *p* < 0.001, Exp(B) = 0.15] suggest that access to resources enabling active participation (e.g., convenient transport, quality healthcare) supports life satisfaction—paralleling evidence that engaged older adults report higher well-being. Finally, echoing Socio-ecological models that integrate physical and social environments, neighborhood environment satisfaction [Wald *χ*^2^ = 187.35, *p* < 0.001, Exp(B) = 0.04] and elder-care service satisfaction [Wald *χ*^2^ = 39.23, *p* < 0.001, Exp(B) = 0.41] were significant predictors. Those “very satisfied” with their neighborhood had only 0.04 times the odds of low life satisfaction, reflecting how physical environments (and associated services like elder care) shape well-being. In summary, while socio-economic factors like education and age showed limited effects, social support (family harmony), activity-enabling resources (transport, healthcare), and socio-ecological factors (neighborhood, elder-care services) emerged as key drivers of life satisfaction, aligning with and contextualizing the four theoretical frameworks within the Chinese older adult population (Table 12).

**Table 12:** Regression analysis of model I (*n* = 2,225).

Predictor	Wald *χ*^2^	df	Overall significance	*B*	Exp(B)
Intercept	6.90	1	**	−6.08**	0.002
Gender	1.90	1	No	0.17	1.19
Age group	2.82	5	No	0.23	1.26
Marital status	5.25	3	No	1.19*	3.27
Education level	1.42	6	No	0.53	1.69
Transport convenience satisfaction	56.65	4	***	−1.67**	0.19
Family harmony satisfaction	187.21	4	***	−3.98**	0.02
Elder-care service satisfaction	39.23	4	***	−0.88	0.41
Medical service satisfaction	57.22	4	***	−1.88*	0.15
Neighborhood environment satisfaction	187.35	4	***	−3.33***	0.04

*p* < 0.05 (marked as *), *p* < 0.01 (marked as **), and *p* < 0.001 (marked as ***). The odds ratio of the first category relative to the last category is presented. Non-significant *p*-values (*p* > 0.05) indicate no statistically significant association with the dependent variable, and that the reported odds ratios should be interpreted with caution.

We then examined how 28 detailed indicators affect overall life satisfaction (Model II). Similarly, since the last option in these four variables—Financial support from children, Emotional support from children, Timeliness of community support, and Proactivity of community care—is “never encountered or does not wish to be disturbed,” we assign values based on their similarity in evaluation to the other options. As it is presented in Table 11, Model II demonstrates a good fit to the data as indicated by its statistical indices. The chi-square statistic for Model II is 1214.792 with 100 degrees of freedom, which is significantly lower than that of Model I (1985.924 with 35 degrees of freedom), suggesting a better fit relative to the number of parameters estimated.

Table 13 summarizes the Wald tests and contrasts the most versus least favorable category for each predictor. Below, we analyze significant variables in ascending order of Exp(B) [since Exp(B) < 1 indicates lower “very dissatisfied” probability than the reference category; smaller values mean lower “very dissatisfied” risk, i.e., higher satisfaction]. Below interprets the regression results through the lens of four core theoretical frameworks—Socio-economic Status Theory, Social Support Theory, Activity Theory, and Socio-ecological Model—to clarify how empirical findings align with theoretical propositions.

**Table 13:** Regression analysis of model II (*n* = 2,225).

Predictor	Wald *χ*^2^	df	Overall significance	*B*	Exp(B)
Gender	1.33	1	No	0.13	1.14
Age group	7.49	5	No	0.22	1.24
Marital status	6.51	3	No	0.50	1.65
Education level	8.06	6	No	−0.28	0.75
Labor income as main source	0.87	1	No	−0.19	0.83
Stable monthly income	2.18	6	No	0.14	1.15
Monthly expenditure on food, etc.	7.65	6	No	0.90	2.46
Healthcare expenditure	18.01	6	**	0.45	0.66
Monthly expenditure on leisure, etc.	3.28	5	No	−0.28	0.75
Living arrangement	3.36	4	No	−0.88	0.41
Per capita living space	17.36	4	**	−0.23	0.80
Self-rated Health	156.84	4	***	−2.21***	0.11
Daily living ability	5.97	3	No	0.47	1.60
Presence of chronic diseases	4.29	1	*	0.26*	1.30
Regular physical check-ups	1.09	3	No	−0.07	0.93
Forgetfulness about medicine	17.18	3	***	0.45*	1.57
Daily physical/mental state	15.57	4	**	−0.49	0.61
None/seldom use any smart devices	0.12	1	No	−0.07	0.94
Frequency of watching short videos	1.78	3	No	0.04	1.04
Participation in physical exercises	7.06	4	No	−0.19	0.83
Participation in community activities	2.34	3	No	−0.03	0.97
Social activities with friends	2.69	3	No	−0.36	0.70
Frequency of traveling outside Xiamen	11.97	5	*	−0.39	0.68
Frequency of contacting children	7.97	4	No	−0.03	0.97
Financial support from children	11.84	3	**	−0.29	0.75
Emotional support from children	38.928	3	0	Yes	1.333
Timeliness of community support	67.884	3	0	Yes	0.172
Proactivity of community care	67.913	3	0	Yes	0.152

*p* < 0.05 (marked as *), *p* < 0.01 (marked as **), and *p* < 0.001 (marked as ***). The odds ratio of the first category relative to the last category is presented. Non-significant *p*-values (*p* > 0.05) indicate no statistically significant association with the dependent variable, and that the reported odds ratios should be interpreted with caution.

From the perspective of Socio-economic Status Theory, which highlights income, education, and occupational prestige as key determinants via material resources and health service access, the regression results reveal nuanced alignment. Healthcare expenditure [Wald *χ*^2^ = 18.01, df = 6, *p* < 0.01, Exp(B) = 0.66] and per capita living space [Wald *χ*^2^ = 17.36, df = 4, *p* < 0.01, Exp(B) = 0.80] emerged as significant predictors, reflecting the theory’s emphasis on material and health-related resources. Notably, stable monthly income (Wald *χ*^2^ = 2.18, df = 6, *p* > 0.05) and education level (Wald *χ*^2^ = 8.06, df = 6, *p* > 0.05) were non-significant, consistent with the Shanghai study finding that absolute income only matters when basic needs are unmet—suggesting the sample’s basic material security weakens the direct effects of traditional socio-economic factors.

Social Support Theory, which distinguishes structural and functional support, is strongly validated by the results. Emotional support from children [Wald *χ*^2^ = 38.93, df = 3, *p* < 0.001, Exp(B) = 1.33] and financial support from children [Wald *χ*^2^ = 11.84, df = 3, *p* < 0.01, Exp(B) = 0.75] highlight the primacy of family support in collectivist contexts, aligning with Shen et al.’s (15) research. Additionally, timeliness of community support [Wald *χ*^2^ = 67.88, df = 3, *p* < 0.001, Exp(B) = 0.17] and proactivity of community care [Wald *χ*^2^ = 67.91, df = 3, *p* < 0.001, Exp(B) = 0.15] were highly significant, extending the theory by demonstrating that formal community support complements family support as a critical protective factor. Interestingly, frequency of contacting children (Wald *χ*^2^ = 7.97, df = 4, *p* > 0.05) was non-significant, suggesting support quality outweighs mere contact frequency.

Activity Theory, which posits meaningful engagement buffers age-related losses, is reflected in specific activity-related predictors. Forgetfulness about medicine [Wald *χ*^2^ = 17.18, df = 3, *p* < 0.001, Exp(B) = 1.57] indirectly reflects the theory—poor medication adherence indicates reduced health management engagement, lowering well-being—while frequency of traveling outside Xiamen [Wald *χ*^2^ = 11.97, df = 5, *p* < 0.05, Exp(B) = 0.68] confirms leisure activities as meaningful contributors. However, participation in physical exercises (Wald *χ*^2^ = 7.06, df = 4, *p* > 0.05) and community activities (Wald *χ*^2^ = 2.34, df = 3, *p* > 0.05) were non-significant, implying only activities perceived as meaningful (e.g., travel) or health-critical (e.g., medication management) exert significant effects.

The Socio-ecological Model, integrating physical and social environments, is strongly supported by the most impactful predictors. Self-rated health [Wald *χ*^2^ = 156.84, df = 4, *p* < 0.001, Exp(B) = 0.11] emerged as the key determinant, reflecting the model’s focus on individual health as a foundational ecological factor, while presence of chronic diseases [Wald *χ*^2^ = 4.29, df = 1, *p* < 0.05, Exp(B) = 1.30] and daily physical/mental state [Wald *χ*^2^ = 15.57, df = 4, *p* < 0.01, Exp(B) = 0.61] further confirm the interplay of physical and psychological domains. Combined with the significant effects of community support factors, these results validate the model’s holistic perspective that individual health and social-environmental contexts collectively shape life satisfaction.

Overall, the regression results underscore the explanatory power of the four frameworks, with socio-ecological factors (especially self-rated health) and social support (family and community) as the most impactful predictors, followed by socio-economic indicators and activity-related factors. These findings highlight that older adults life satisfaction stems from the interplay of individual, socio-economic, social support, and environmental factors, emphasizing the need for holistic interventions across multiple domains.

## 5 Discussion

The study reveals that the overall life satisfaction among the older adults in Xiamen is relatively high, with 91.5% of respondents reporting being either very satisfied or fairly satisfied. Key factors significantly influencing life satisfaction include health status, social support, and economic security. Specifically, self-rated health and daily physical and mental state are crucial health-related predictors. Social support from the community, such as timely help and proactive care, plays a significant role in enhancing life satisfaction. Economic factors like healthcare expenditure and financial support from children also contribute to higher satisfaction levels. Additionally, from an aggregated perspective, satisfaction with family harmony, transport convenience, medical services, neighborhood environment, and older adults care services are identified as important determinants of overall life satisfaction.

To further synthesize the four guiding theoretical frameworks, we focus on their complementary mechanisms in shaping older adults life satisfaction: the Socio-ecological Model serves as the overarching lens, emphasizing the dynamic interplay between individual and contextual factors that unifies the other three theories, with individual-level factors (e.g., activity engagement from Activity Theory, material resources from Socio-economic Status Theory) interacting with social support networks (Social Support Theory) and environmental contexts (e.g., community services) to influence life satisfaction; empirically, this synthesis is reflected in the intersecting and compensatory effects of key factors, such as sufficient material resources facilitating meaningful activities while formal community support compensates for inadequate family financial support to enhance satisfaction, and strong social support mitigating the negative impact of limited economic resources as active daily participation buffers well-being decline from chronic diseases; this integrated perspective reveals that older adults life satisfaction is a multifaceted outcome shaped by the confluence of material resources, social connections, individual activity, and contexts, moving beyond fragmented single-factor explanations to provide a more comprehensive theoretical basis for age-friendly policies and guide future research to adopt integrated theoretical approaches.

The findings align with global and Chinese empirical studies that consistently identify health and social support as pivotal predictors of older adults life satisfaction. For instance, existing research has underscored self-rated health and functional independence as strong correlates of life satisfaction (11, 23), while social support from family and community has been repeatedly confirmed as a central determinant across contexts (5, 15, 26). Unique to the urban setting of Xiamen, this study reveals a more pronounced impact of community services compared to other regions, likely attributed to the city’s advanced urban infrastructure and robust social welfare policies. Additionally, the influence of Xiamen’s specific pension policies—coupled with stable monthly income and financial support from children—further emphasizes the enduring role of economic security in enhancing the older adults’s life satisfaction.

Beyond corroborating prior research, this study makes meaningful theoretical contributions. With 91.5% of respondents reporting relatively high life satisfaction, it sheds light on regional variations in older adults well-being within China, highlighting the unique interplay of community services and local pension policies in urban contexts. It enriches the application of Social Support Theory in urban Chinese settings by demonstrating that both family support (emotional and financial) and formal community support exert significant, complementary effects on life satisfaction—extending the theory’s focus beyond informal ties to incorporate formal social systems. The findings also reinforce Socio-ecological models, which integrate physical and social neighborhood environments as distal determinants of life satisfaction (5, 10, 16, 24), as evidenced by the significant impacts of community care proactivity, healthcare accessibility, and living conditions.

Practically, the results offer actionable insights for policymakers in Xiamen and similar urban areas. To enhance older adults life satisfaction, interventions should prioritize three key areas: improving healthcare accessibility, strengthening community-based older adults support systems, and optimizing financial security mechanisms. Specific recommendations include expanding home care services (e.g., home-based medical care, daily assistance), upgrading public infrastructure (such as barrier-free renovations in historical blocks to facilitate mobility), and refining pension policies to ensure stable and adequate financial support. These targeted measures can foster a more supportive, age-friendly environment, ultimately promoting the overall well-being and quality of life of the older adults population.

## 6 Conclusion

This study set out to investigate the life satisfaction of the older adults in Xiamen, China, and identify key factors influencing their subjective well-being. Adopting a quantitative, cross-sectional survey design, we collected data from 2,225 community-dwelling older residents across urban and rural areas of Xiamen between March and May 2025. The questionnaire covered a comprehensive range of domains, including demographics, economic status, housing and environment, health, social and activity participation, social support, and life satisfaction.

Our key findings highlight that the majority of Xiamen’s older population reports relatively high levels of life satisfaction. However, significant disparities exist in health status, social participation, and economic security among the older adults. Health-related factors, such as self-rated health and daily physical and mental state, emerged as crucial predictors of life satisfaction. Social support from both family and community, particularly timely help and proactive care from community services, also played a significant role in enhancing life satisfaction. Economic factors, including stable monthly income, healthcare expenditure, and financial support from children, were identified as important determinants.

The study underscores the importance of addressing the health, social, and economic needs of the older adults to enhance their life satisfaction in Xiamen. Improving healthcare accessibility, enhancing community-based older adults, activities, and strengthening financial support systems are essential steps for policymakers. These interventions can create a more supportive environment for the older adults, promoting their overall well-being and quality of life.

The value of this study lies in its contribution to the understanding of regional variations in older adults, life satisfaction within China and its practical implications for local policymakers. By highlighting the unique role of community services and specific pension policies in an urban context like Xiamen, this research provides actionable insights that can be applied to other rapidly aging Chinese cities with similar development levels and demographic structures. Future research should continue to explore these dynamics longitudinally and include a broader range of older adults populations to deepen our understanding of how to best support the well-being of the older adults in China.

The study has several limitations. First, the cross-sectional design limits the ability to establish causality between the identified factors and life satisfaction. Future research should consider longitudinal studies to explore these relationships over time. Second, there is potential sampling bias, as the study may underrepresent institutionalized older adults individuals. This group may have different experiences and needs compared to community-dwelling older. Third, the data are based on self-reports, which may introduce response bias. Future studies could benefit from incorporating objective measures of health and social support to complement self-reported data. Fourth, the measurement of life satisfaction relies on a single Likert-scale item, which may capture only a broad overview of subjective well-being and overlook nuanced differences across life domains. Future research should adopt more detailed, multi-item scales (e.g., the Satisfaction with Life Scale, SWLS) or domain-specific measures to cross-validate the findings and enhance the depth of measurement.

## Data Availability

The original contributions presented in the study are included in the article/Supplementary material, further inquiries can be directed to the corresponding author.
