# Supplementary material for: Life satisfaction of older adults and its influencing factors: an exploratory study of community-dwelling older adults in Xiamen, China
Source: Front Public Health. 2026 Jan 30;14:1699050. doi: 10.3389/fpubh.2026.1699050 (PMC12900670; doi:10.3389/fpubh.2026.1699050)
Supplement: Supplementary file 1 [file Data_Sheet_1.docx]

# ****Questionnaire on the Living Conditions of Older Adults in Xiamen, 2025****

To gain an in-depth understanding of the living conditions of older adults in Xiamen, and to ensure that the city’s aging policies better meet their actual needs, the Municipal Office on Aging has designed this questionnaire. All information you provide will be kept strictly confidential and used solely for research purposes by relevant authorities. Thank you for your support and cooperation!

## ****Section 1: Basic Information****

****1.Gender:****
☐ Male
☐ Female

****2.Age:****
☐ 60–64
☐ 65–69
☐ 70–74
☐ 75–79
☐ 80–84
☐ 85 and above

****3.Marital Status:****
☐ Married
☐ Widowed
☐ Divorced
☐ Never married

****4.Education Level:****
☐ No formal education
☐ Primary school
☐ Middle school
☐ High school / Technical secondary school
☐ Associate degree
☐ Bachelor’s degree
☐ Master’s degree or above

## ****Section 2: Economic Status****

****5.Main sources of income (multiple choices allowed):****
☐ Pension
☐ Labor income
☐ Financial support from children
☐ Savings or interest
☐ Investment, financial products, or stock returns
☐ Government subsidies
☐ Other (please specify): ___________

****6.Approximate monthly stable income (e.g., pension, interest; excluding risky or unstable income such as stocks):****
☐ Under ¥500
☐ ¥501–1,000
☐ ¥1,001–2,000
☐ ¥2,001–3,000
☐ ¥3,001–5,000
☐ ¥5,001–8,000
☐ Over ¥8,001

****7.Approximate monthly personal expenses on food, clothing, housing, and transportation:****
☐ Under ¥500
☐ ¥501–1,000
☐ ¥1,001–2,000
☐ ¥2,001–3,000
☐ ¥3,001–5,000
☐ ¥5,001–8,000
☐ Over ¥8,001

****8.Approximate monthly personal medical and health expenses:****
☐ Under ¥500
☐ ¥501–1,000
☐ ¥1,001–2,000
☐ ¥2,001–3,000
☐ ¥3,001–5,000
☐ ¥5,001–8,000
☐ Over ¥8,001

****9.Approximate monthly spending on leisure, tourism, culture, sports, education, etc.:****
☐ Under ¥500
☐ ¥501–1,000
☐ ¥1,001–2,000
☐ ¥2,001–3,000
☐ ¥3,001–5,000
☐ Over ¥5,001

## ****Section 3: Living Conditions****

****10.Current living arrangement:****
☐ Living alone
☐ Living with spouse
☐ Living with children
☐ Living with relatives, friends, or caregivers
☐ Living in a nursing home

****11.Approximate per capita living space in your household:****
☐ Over 40 m²
☐ 30–40 m²
☐ 20–30 m²
☐ 10–20 m²
☐ Under 10 m²

****12.Satisfaction with your community/village living environment (including hygiene, sunlight, noise, scenery, shopping, transportation, utilities, etc.):****
☐ Very satisfied
☐ Somewhat satisfied
☐ Neutral
☐ Somewhat dissatisfied
☐ Very dissatisfied

## ****Section 4: Health Status****

****13.Self-rated health status:****
☐ Very good
☐ Good
☐ Fair
☐ Poor
☐ Very poor

****14.Ability to perform daily activities:****
☐ Fully independent
☐ Occasionally need help
☐ Often need help
☐ Completely dependent or severely need help

****15.Do you have any chronic diseases? (multiple choices allowed):****
☐ Cardiovascular diseases (e.g., hypertension, coronary heart disease, heart failure)
☐ Metabolic diseases (e.g., diabetes, hyperlipidemia)
☐ Bone and joint diseases (e.g., osteoporosis, arthritis)
☐ Neurological diseases (e.g., Alzheimer’s, Parkinson’s)
☐ Respiratory diseases (e.g., COPD, pulmonary fibrosis)
☐ Urinary system diseases (e.g., chronic kidney disease, prostate enlargement)
☐ Vision-related diseases (e.g., cataracts, macular degeneration)
☐ Other chronic diseases
☐ No chronic diseases

****16.Do you undergo regular health checkups?****
☐ At least once a year
☐ Once every 1–2 years
☐ Once every 3–5 years
☐ Not in the past 5 years

****17.When you need to take medication (short-term or long-term), do you often forget to take it on time?****
☐ Often forget
☐ Occasionally forget
☐ Rarely forget
☐ Never forget

****18.How would you describe your daily physical and mental state?****
☐ Energetic and happy, always enthusiastic about life
☐ Generally positive, enjoy life, but occasionally feel tired
☐ Emotionally stable, sometimes feel bored or unmotivated
☐ Often feel tired or down, lack interest in new things, worry about the future
☐ Always feel exhausted or depressed, strong sense of loneliness, lack of interest in life

## ****Section 5: Lifestyle & Social Habits****

****19.Do you own and regularly use any of the following smart devices? (multiple choices allowed):****
☐ Smartphone
☐ Tablet
☐ Desktop or laptop
☐ Smartwatch or fitness tracker
☐ Other smart wearables
☐ None of the above / rarely use

****20.Do you often watch short videos online (e.g., Douyin, Kuaishou, Tencent Video)?****
☐ Watch every day
☐ Often watch
☐ Occasionally watch
☐ Rarely watch

****21.Do you regularly participate in physical exercise (e.g., brisk walking, running, aerobics, ball games, dancing)?****
☐ Almost every day
☐ 2–3 times a week
☐ Once a week
☐ 1–2 times a month
☐ Almost never

****22.Do you often participate in community-organized activities (e.g., dancing, singing, ball games, martial arts, gatherings, performances)?****
☐ Often participate
☐ Occasionally participate
☐ Rarely participate
☐ Never participate

****23.Do you often join friends in activities (e.g., dancing, singing, ball games, martial arts, gatherings, performances), or participate in such activities organized by senior universities, associations, or nursing homes?****
☐ Often participate
☐ Occasionally participate
☐ Rarely participate
☐ Almost never

****24.Do you often travel outside Xiamen for family visits, sightseeing, tourism, or leisure?****
☐ Frequently (more than 6 times a year)
☐ 5–6 times a year
☐ 3–4 times a year
☐ 1–2 times a year
☐ Occasionally
☐ Almost never

## ****Section 6: Family & Social Support****

****25.How often do you contact your children?****
☐ Live together or contact daily
☐ Contact 1–2 times a week
☐ Contact 1–2 times a month
☐ Contact a few times a year
☐ Almost no contact

****26.When you need financial help, do you receive timely support from your children?****
☐ Often receive strong support
☐ Occasionally receive moderate support
☐ Rarely receive limited support
☐ Almost no support
☐ Never encountered this situation

****27.When you need emotional support or comfort, do you receive timely support from your children?****
☐ Often receive strong support
☐ Occasionally receive moderate support
☐ Rarely receive limited support
☐ Almost no support
☐ Never encountered this situation

****28.When you seek help from your community/village, do you receive timely support?****
☐ Often receive help
☐ Occasionally receive help
☐ Rarely receive help
☐ Almost never
☐ Never encountered this situation

****29.Does your community/village proactively check on your well-being via visits, calls, or WeChat?****
☐ Almost every month
☐ 3–5 times a year
☐ 1–2 times a year
☐ Almost never
☐ I prefer not to be disturbed

## ****Section 7: Life Satisfaction****

****30.Overall satisfaction with living in Xiamen:****
☐ Very satisfied
☐ Somewhat satisfied
☐ Neutral
☐ Somewhat dissatisfied
☐ Very dissatisfied

****31.Satisfaction with family harmony and children’s support:****
☐ Very satisfied
☐ Somewhat satisfied
☐ Neutral
☐ Somewhat dissatisfied
☐ Very dissatisfied

****32.Satisfaction with daily transportation convenience:****
☐ Very convenient
☐ Somewhat convenient
☐ Neutral
☐ Somewhat inconvenient
☐ Very inconvenient

****33.Satisfaction with elderly care services (meal assistance, cleaning, bathing, mobility support, etc.):****
☐ Very satisfied
☐ Somewhat satisfied
☐ Neutral
☐ Somewhat dissatisfied
☐ Very dissatisfied
☐ Not familiar

****34.Satisfaction with medical services (registration, consultation, prescriptions, treatment, etc.):****
☐ Very satisfied
☐ Somewhat satisfied
☐ Neutral
☐ Somewhat dissatisfied
☐ Very dissatisfied

****End of Questionnaire****

Thank you for taking the time to complete this survey! Your input is invaluable and will help the government develop better policies to improve the quality of life for older adults. Thank you again for your support and cooperation!
